# Repurposing Tranexamic Acid as an Anticancer Agent

**DOI:** 10.3389/fphar.2021.792600

**Published:** 2022-01-13

**Authors:** Mary E. Law, Bradley J. Davis, Amanda F. Ghilardi, Elham Yaaghubi, Zaafir M. Dulloo, Mengxiong Wang, Olga A. Guryanova, Coy D. Heldermon, Stephan C. Jahn, Ronald K. Castellano, Brian K. Law

**Affiliations:** ^1^ Department of Pharmacology and Therapeutics, University of Florida, Gainesville, FL, United States; ^2^ Department of Chemistry, University of Florida, Gainesville, FL, United States; ^3^ UF Health Cancer Center, University of Florida, Gainesville, FL, United States; ^4^ Department of Medicine, University of Florida, Gainesville, FL, United States

**Keywords:** MYC, S6K1, CDCP1, STAT3, protein synthesis, acetylation, tranexamic acid, cancer

## Abstract

Tranexamic Acid (TA) is a clinically used antifibrinolytic agent that acts as a Lys mimetic to block binding of Plasminogen with Plasminogen activators, preventing conversion of Plasminogen to its proteolytically activated form, Plasmin. Previous studies suggested that TA may exhibit anticancer activity by blockade of extracellular Plasmin formation. Plasmin-mediated cleavage of the CDCP1 protein may increase its oncogenic functions through several downstream pathways. Results presented herein demonstrate that TA blocks Plasmin-mediated excision of the extracellular domain of the oncoprotein CDCP1. *In vitro* studies indicate that TA reduces the viability of a broad array of human and murine cancer cell lines, and breast tumor growth studies demonstrate that TA reduces cancer growth *in vivo*. Based on the ability of TA to mimic Lys and Arg, we hypothesized that TA may perturb multiple processes that involve Lys/Arg-rich protein sequences, and that TA may alter intracellular signaling pathways in addition to blocking extracellular Plasmin production. Indeed, TA-mediated suppression of tumor cell viability is associated with multiple biochemical actions, including inhibition of protein synthesis, reduced activating phosphorylation of STAT3 and S6K1, decreased expression of the MYC oncoprotein, and suppression of Lys acetylation. Further, TA inhibited uptake of Lys and Arg by cancer cells. These findings suggest that TA or TA analogs may serve as lead compounds and inspire the production of new classes of anticancer agents that function by mimicking Lys and Arg.

## Introduction

Repurposing FDA-approved drugs as anticancer agents represents a powerful method for rapidly advancing new cancer therapeutics into the clinic especially since many of the safety issues are already established. This is particularly true for a drug like Tranexamic Acid (TA), which is on The WHO List of Essential Medicines and has an excellent safety record while being widely used for over 50 years in a broad range of formulations and dosages in numerous different indications. TA is clinically used to suppress hemorrhaging by blocking Plasminogen conversion to Plasmin. TA functions as a Lys side chain mimetic and prevents Plasmin formation by occupying Lys binding sites on Plasminogen (Iwamoto 1975). Due to its ability to mimic the side chains of Lys, and perhaps Arg, yet not being incorporated into protein due to its structure, TA has the potential to alter many biological processes. For instance, basic amino acid residues are present in the nuclear import signals of proteins (Laskey et al., 1996) and in the consensus recognition sites of many protein kinases, most noteworthy, the AGC family of serine/threonine kinases (Zhang et al., 2002). Additionally, Lys side chains in proteins are subject to multiple posttranslational modifications that include acetylation, methylation, ubiquitination, and SUMOylation. The “histone code” of epigenetic regulation involves Lys and Arg modifications (Turner 2000; Izzo and Schneider 2010; Tweedie-Cullen et al., 2012; Blee et al., 2015). Given the important role of Lys and its modifications in the control of multiple cellular processes, we hypothesized that in addition to its widespread use to suppress bleeding, TA may inhibit the enzymes that carry out Lys modification in proteins in a competitive manner, suppress the function of Lys/Arg-rich protein-protein recognition motifs, or compete with the transporters that mediate Lys/Arg uptake.

While TA has been used to manage cancer and cancer treatment-associated sequela in patients (Soma et al., 1980; Kikuchi et al., 1986; Oertli et al., 1994; Wu et al., 2006; Longo et al., 2018; Lohani et al., 2020; Jaffer et al., 2021; Lohani et al., 2021), it has never been used clinically as an anti-cancer therapeutic, and considerations of TA anticancer actions have focused on its blockade of Plasmin production. The goals of the studies carried out herein were three-fold: 1. To examine the impact of TA treatment on the viability of cancer cells to evaluate the possibility of repurposing TA as an anticancer agent, 2. To determine if TA may provide a lead compound to serve as the basis of derivatives with greater anticancer efficacy and potency, and enhanced selectivity for specific downstream responses, and 3. Because of its unusual mechanism of action, test whether TA may prove useful in combination anticancer regimens. Given our previous work with a class of anticancer compounds termed Disulfide bond Disrupting Agents (DDAs) (Ferreira et al., 2015; Ferreira et al., 2017; Besse et al., 2019; Wang et al., 2019a; Wang et al., 2019b) and observations below that TA and DDAs have partially overlapping biochemical mechanisms of action, we evaluated the anticancer activity of TA and DDA mono- and combination therapies in an animal model of breast cancer. The results show that TA suppresses the viability of a broad array of human and murine cancer cell lines, exhibits previously unreported effects on cell signaling pathways that control cancer cell proliferation and oncogenic transformation, and modulates the acetylation status of Lys residues in a subset of acetylated proteins. TA and the DDAs tcyDTDO and dMtcyDTDO strongly suppress the growth of breast tumors individually and may be useful in combination.

## Materials and Methods

### MTT Cell Viability Assays

MTT cell viability assays were performed by plating cells at 7,500/well in 96-well plates, followed by treatment of the cells with increasing concentrations of TA (Chem-Impex International, Inc., Wood Dale, IL, United States) for 72 h at 37°C. After removal of the cell media, cells were incubated with 0.5 mg/ml MTT (3-(4,5-dimethylthiazol-2-yl)-2,5-diphenyltetrazolium bromide) (Biomatik, Wilmington, DE, United States) in PBS for 3 h. The MTT solution was subsequently removed and the MTT formazan product was dissolved in 100 μl of DMSO. Absorbance (570 and 690 nm) of the MTT formazan product was measured in a plate reader.

### Tumor Studies

**FIGURE 1:**
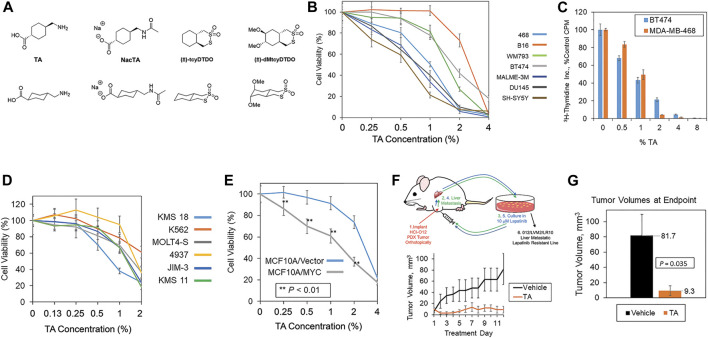
TA decreases cancer cell viability and breast tumor growth. **(A)** Chemical structures of TA, *N*-acetylated TA (NacTA), and the DDAs tcyDTDO and dMtcyDTDO. **(B)** The indicated panel of cancer cell lines were treated for 72 h with different TA concentrations and cell viability was measured using the MTT assay. Results are the average of six replicates and presented as the average ±S.D. These findings are representative of three biological replicates with similar results. **(C)** MDA-MB-468 and BT474 cells were treated for 24 h with the indicated concentrations of TA and the incorporation of tritiated thymidine into DNA was evaluated. Results are the average of six replicates and presented as the average ±S.D. These findings are representative of three biological replicates with similar results. **(D)** Effects of TA on the viability of a panel of leukemia, lymphoma, and multiple myeloma cell lines. Results are the average of six replicates and presented as the average ±S.D. These findings are representative of three biological replicates with similar results. **(E)** Cell viability assay showing that TA more potently reduces the viability of MCF10A mammary epithelial cells overexpressing MYC compared with the vector control line. Statistically significant differences between the lines were observed at 0.25, 0.5, 1, and 2% TA. *p*-values were calculated using unpaired Student’s *t*-tests. Results are the average of six replicates and presented as the average ±S.D. These findings are representative of three biological replicates with similar results. **(F)** Tumor growth study in which mice bearing orthotopic 012/LVM2/LR10 mammary tumors were treated once daily with 375 mg/kg TA. The upper panel illustrates how the 012/LVM2/LR10 cancer model was derived. Tumor dimensions were measured daily and tumor volumes calculated. Averages of within-group tumor volumes are presented and error bars represent standard error of the mean. **(G)** Tumor volumes measured at day 12 exhibit a statistically significant TA-mediated reduction in tumor volume (*p* = 0.035), with an approximately nine-fold smaller tumor volume in the TA group than the vehicle group. *p*-values were calculated using an unpaired Student’s *t*-test. Each treatment group contained five animals.

012/LVM2/LR10 tumors were generated by injection of 1 × 10^6^ 012/LVM2/LR10 cells into the #4 mammary fat pads of NOD-SCID-gamma (NSG) mice from Jackson Laboratories (Bar Harbor, ME, United States), as described previously (Wang et al., 2019b). In the tumor study in Figures 1F,G, mice bearing HCI-012/LVM2/LR10 breast tumors were treated once daily with 375 mg/kg TA by oral gavage for 12 days followed by breast tumor removal. In the tumor study in Figure 4A, tumors were generated using the 012/LVM2/LR10 cancer cell model as above and the animals were treated with vehicle (peanut oil), 375 mg/kg TA in water, 10 mg/kg dMtcyDTDO in peanut oil, or 375 mg/kg TA + 10 mg/kg dMtcyDTDO once daily by oral gavage. Tumor dimensions were recorded daily at the time of treatment.

### Protein Synthesis Assays

Protein synthesis assays were performed by measurement of the incorporation of ^3^H-Leucine (Perkin Elmer, Waltham, MA, United States) into protein, as detailed previously (Law et al., 2000a; Law et al., 2000b).

### Thymidine Incorporation Assays

Thymidine incorporation assays were described in a previous publication (Corsino et al., 2009).

### Cellular Uptake of Lys and Arg

MDA-MB-468 cells were plated at 10^5^/well in 24 well plates and incubated overnight to permit attachment. Cells were gently washed with PBS and fed with serum-free and Arg and Lys-free DMEM (Thermo Fisher, Waltham, MA, United States, Cat. # 88364) containing the indicated concentrations of TA and incubated for 24 h. Cells were pulsed for 2 h with 0.5 μCi/well ^3^H-Arg (PerkinElmer, Waltham, MA, United States, Cat. # NET1123250UC) or 0.2 μCi/well ^14^C-Lys (PerkinElmer, Cat. # NEC280E050UC). The cells were washed twice with PBS and lysed in 300 μl/well 0.1% SDS in 0.5 N NaOH. Uptake of radiolabeled amino acids was quantified by counting 100 μl of the lysate from each well in a scintillation counter.

### P300 Acetylation Assays

P300 Acetylation assays were performed by incubating the reaction buffer (50 mM Tris, pH 8.0, 10% glycerol, 0.1 mM EDTA, and 1 mM DTT), 0.5 μg p300 (ENZO Life Sciences, Farmingdale, NY, United States), 0.5 μg Histone H3 (NEB, Ipswich, MA, United States), and 60 μM Acetyl CoA (Sigma-Aldrich, St. Louis, MO, United States) in the presence or absence of TA, NacTA, or tcyDTDO for 2 h at 30°C. Reactions were terminated by the addition of 2X SDS sample buffer and boiling for 10 min, followed by immunoblot analysis employing a 45 min transfer to 0.2 μm nitrocellulose using CAPS buffer, pH 11.

### NacTA Synthesis

#### General Methods

Reagents and solvents were purchased from commercial sources without further purification unless otherwise specified. ^1^H and ^13^C NMR spectra were recorded using commercially obtained D_2_O (Cambridge Isotope Laboratories, Inc.) on a Bruker 600 spectrometer (^1^H at 600 MHz) and Bruker 400 spectrometer (^13^C at 101 MHz), respectively. Chemical shifts (*δ*) are given in parts per million (ppm) relative to TMS and referenced to residual protonated D_2_O (*δ*H = 4.79 ppm). Coupling constants (*J*) are given in Hz; spin multiplicities are presented by the following symbols: s (singlet), bs (broad singlet), d (doublet), t (triplet), q (quartet), and m (multiplet). Electrospray ionization (ESI) high resolution mass spectra (HRMS) were recorded on an Agilent 6200 ESI-TOF instrument operating in positive or negative ion mode as stated.

#### Synthesis of Sodium (1*r*,4*r*)-4-(Acetamidomethyl)Cyclohexane-1-Carboxylate, NacTA

The preparation of NacTA was adapted from a procedure reported by Kusakabe et al. (2010). Tranexamic acid (1.90 g, 12.1 mmol) was suspended in acetic anhydride (9.70 ml, 103 mmol) and concentrated H_2_SO_4_ (5.00 μl) was carefully added. The suspension was stirred for 18 h at rt. Water (10.0 ml) was added and the mixture was stirred for 1 h at rt to decompose any remaining acetic anhydride. The solution was concentrated under vacuum, and the residue was further dried with the addition of toluene and subsequent removal of solvent under reduced pressure. The resulting precipitate was collected by vacuum filtration and washed with ethyl ether to give NacTA as a white powder. For the sodium salt preparation, NacTA was suspended in water and an aqueous solution of 2 M NaOH was added with stirring at rt until the pH increased to 13. The suspension was then stirred for an additional 1 h at rt until the entire solid dissolved. The solvent was then evaporated under reduced pressure and the precipitate was recrystallized in ethanol to remove remaining NaOH salt. The white precipitate obtained was then collected by vacuum filtration and washed with ethanol to afford the product as a white solid (1.30 g, 5.80 mmol, 48% yield). ^1^H NMR (600 MHz, D_2_O) *δ* 3.01 (d, *J* = 6.8 Hz, 2H), 2.08 (tt, *J* = 12.2, 3.5 Hz, 1H), 1.98 (s, 3H), 1.90–1.84 (m, 2H), 1.77 (dd, *J* = 13.2, 3.6 Hz, 2H), 1.52–1.42 (m, 1H), 1.31 (qd, *J* = 13.0, 3.7 Hz, 2H), 0.96 (qd, *J* = 12.8, 3.6 Hz, 2H). The NH proton was not observed. ^13^C NMR (101 MHz, D_2_O) *δ* 186.50, 173.97, 46.92, 45.53, 36.78, 29.49, 29.23, 21.80. HRMS-ESI: *m*/*z* [M]^–^ calcd for [C_10_H_16_NO_3_]^–^: 198.1136; found: 198.1148.

### Cell Culture, Preparation of Cell Extracts, and Immunoblot Analysis

The BT474, MDA-MB-231, MDA-MB-468, MCF10A, HMEC, B16, MALME-3M, DU145, SH-SY5Y, K 562, and MOLT4 cells lines were purchased from American Type Culture Collection (ATCC) (Manassas, VA USA). The KMS-11, KMS-18, JIM-3, and 4937 cell lines were kindly provided by Dr. Olga Guryanova, University of Florida Health Cancer Center and the WM793 cell line was generously provided by Dr. W. Douglas Cress, Moffitt Cancer Center. Derivation of the HCI-012/LVM2/LR10 cell line was previously described (Wang et al., 2019a; Wang et al., 2019b). MCF10A cell lines stably expressing MYC were generated as detailed in previous reports (Law et al., 2002; Wang et al., 2019a; Wang et al., 2019b), as was the generation of the 231/E-Cad and 231/E-Cad-GFP cells lines (Law et al., 2013). Cell lysates were prepared, as described previously (Law et al., 2002). The antibodies used for immunoblot analyses are listed in Supplementary Table S1.

### *In Silico* Molecular Docking

Compounds were docked into the Arg binding pocket of the bacterial homolog of CAT (CATh). The crystal structure was downloaded from the RCSB protein databank (Accession # 6f34, (Jungnickel et al., 2018)) in .pdb format and all heteroatoms were removed. The molecular surface of the protein was created using the Chimera program and spheres representing potential docking sites were created using SPHGEN. Spheres that did not correspond to the Arg binding pocket were removed and the SHOWBOX program created a 3-dimensional box around the spheres with a 4 Å buffer on all sides. The GRID program was then used to generate files representing the electrostatic and van der Waals forces produced by the protein in the region of interest. The DOCK program was used to calculate GRID scores for the ligands of interest, consisting of electrostatic and van der Waals interactions, and ranked depending on predicted binding affinity. All programs were from UCSF and, except for Chimera, are included in the DOCK 6.7 suite (Allen et al., 2015). Images were generated using PyMol from Schrodinger.

### Statistical Analysis

#### Statistics

Statistical analysis was performed using the unpaired Student’s *t*-test. Error bars represent standard deviation of the mean unless otherwise indicated and all *p* values are two-tailed. *p* values are documented in either the figures or legends. Points from cell culture studies plotted on bar or line graphs are the average of six or more replicates and are representative of three or more independent experiments. Quantitation of immunoblot results was performed by scanning the blots, inverting the images with Photoshop (San Jose, CA, United States), and quantitating band intensities using ImageJ (NIH). Bar graphs of band intensities are presented as the average and error bars correspond to the standard error of the mean.

## Results

### Tranexamic Acid Suppresses Cancer Cell Viability *In Vitro* and Tumor Growth *In Vivo*

Chemical structures of TA and the other compounds used or mentioned in the work described here are shown in Figure 1A. MTT cell viability assays showed that TA suppressed the viability of a variety of cancer cell lines including melanoma (B16, WM793, and MALME-3M), breast (MDA-MB-468 and BT474), prostate (DU145), and neuroblastoma (SH-SY5Y) lines (Figure 1B). DU145 was one of the more TA-sensitive lines and exhibited statistically significant TA-mediated viability suppression at the lowest TA concentration tested 0.25% (w/v) compared with the vehicle control (Supplementary Figure S1A). Analysis of TA effects on cell proliferation, as measured by ^3^H-thymidine incorporation, showed that TA inhibited DNA synthesis in the BT474 and MDA-MB-468 cells over the same concentration range that it reduces cell viability (Figure 1C). Studies of TA effects on the viability of leukemia, lymphoma, and multiple myeloma lines using the XTT assay showed concentration-dependent reductions in cancer cell viability (Figure 1D). The MYC oncogene is frequently overexpressed in human cancers (Rothberg 1987; Saksela 1990; Shiu et al., 1993). However, MYC overexpression renders cancer cells vulnerable to some therapeutics, resulting in collateral vulnerabilities amenable to synthetic lethal targeting approaches (Hsieh and Dang 2016; Lee et al., 2019). Therefore, we examined if MYC overexpression increased the sensitivity of the MCF10A human breast epithelial cell line to TA. MYC overexpression enhanced the TA suppression of cell viability across an array of TA concentrations suggesting that MYC overexpression may be useful as a marker to identify TA-sensitive tumors (Figure 1E).

We employed a previously described metastatic, Lapatinib-resistant, orthotopic xenograft model, 012/LVM2/LR10 (Wang et al., 2019a; Wang et al., 2019b), to examine if TA reduces the growth of breast tumors in experimental animals (Figure 1F, upper panel). Tumor growth studies revealed that 375 mg/kg TA suppressed the growth of 012/LVM2/LR10 tumors without evidence of treatment toxicity (Figure 1F, lower panel). At the 12-day treatment endpoint, tumors in the TA treatment group were approximately one-ninth the size of the tumors in the vehicle group (Figure 1G).

### TA Suppresses S6K1 and STAT3 Phosphorylation

**FIGURE 2:**
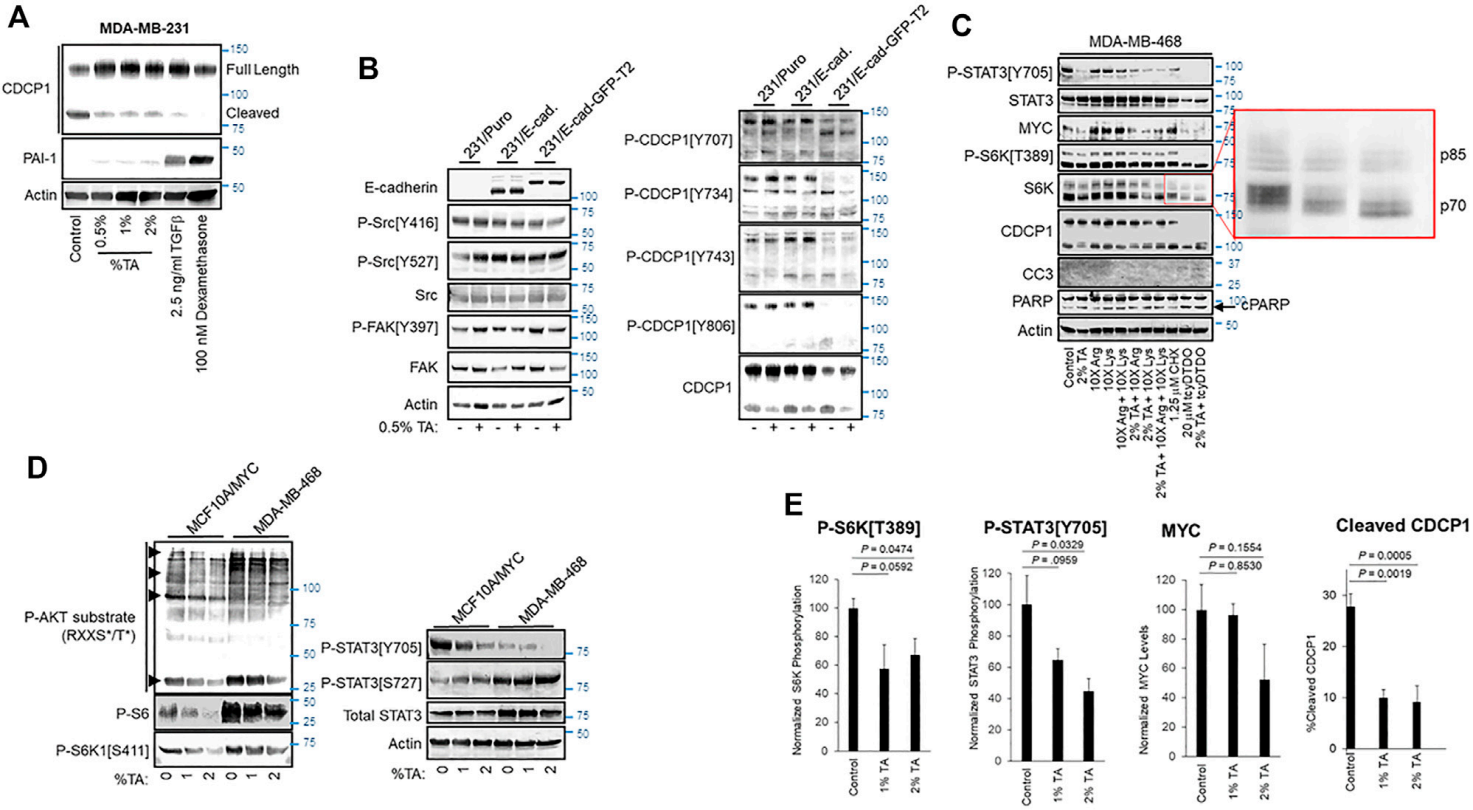
TA blocks CDCP1 cleavage, reduces MYC expression, and decreases STAT3 and S6K1 phosphorylation. **(A)** MDA-MB-231 cells were treated as indicated for 24 h and cell extracts were subjected to immunoblot analysis. **(B)** Vector control MDA-MB-231 cells or cells overexpressing E-cadherin (E-Cad.) or an E-cadherin-Green Fluorescent Protein fusion (E-Cad-GFP) were treated for 24 h with vehicle or 0.5% TA and cell extracts were subjected to immunoblot analysis. **(C)** MDA-MB-468 cells were treated for 24 h as indicated and subjected to immunoblot analysis. As indicated Lys and/or Arg were present in some treatments at 10X the concentration present in DMEM. The expanded red box shows altered mobility due to differential phosphorylation of the p70 and p85 isoforms of S6K1. Note that TA combined with tcyDTDO reduced S6K1 phosphorylation more than that observed with tcyDTDO alone. Inhibition of protein synthesis with 1.25 μM Cycloheximide (CHX) reduced STAT3 tyrosine phosphorylation, but did not alter MYC levels, S6K1 phosphorylation, or CDCP1 cleavage. **(D)** Immunoblot analyses examining the effects of treating MYC overexpressing MCF10A cells or MDA-MB-468 cells for 24 h with 1 or 2% TA on STAT3 Ser and Tyr phosphorylation, S6K1 phosphorylation on Ser^411^, phosphorylation of the S6K1 substrate, S6, and phosphorylation of proteins on consensus phosphorylation sites for AKT. Note that the AKT consensus phosphorylation sequence overlaps with that of S6K1. **(E)** Quantitation of the effects of 24 h treatment of MDA-MB-468 cells with 1% or 2% TA on S6K1 phosphorylation, STAT3 phosphorylation, MYC expression levels, or CDCP1 cleavage across multiple experiments. Results are the average of six replicates and presented as the average ±S.E.M. *p*-values were calculated using an unpaired Student’s *t*-test.

We next sought to examine the mechanisms through which TA impacts tumor growth. TA may exhibit anticancer activity through effects on Plasmin activity and via Plasmin-independent actions (O'Grady et al., 1981; Tanaka et al., 1982; Stonelake et al., 1997; Dunbar et al., 2000; Suojanen et al., 2009). Previous work indicated that Plasmin cleaves the extracellular domain of the transmembrane protein CUB Domain-Containing Protein 1 (CDCP1) to produce a truncated protein that exhibits differential protein-protein interactions and signaling functions compared with the full-length protein, suggesting that cleaved CDCP1 (cCDCP1) may contribute to tumor growth and progression (Casar et al., 2014; Law et al., 2016; Wright et al., 2016). However, the ability of TA to suppress CDCP1 cleavage has not been examined. TA reduced CDCP1 cleavage in MDA-MB-231 breast cancer cells (Figure 2A). Unlike Transforming Growth Factor-β (TGFβ) or the glucocorticoid dexamethasone, which block CDCP1 cleavage by upregulating Plasminogen Activator Inhibitor-1 (PAI-1) (Law et al., 2013; Law et al., 2016), TA did not increase PAI-1 levels (Figure 2A). cCDCP1 binds E-cadherin and interferes with its cell-cell adhesive functions, while the full-length protein does not (Law et al., 2016). Blocking CDCP1 cleavage via TA administration may be useful on restoring the anti-invasive functions of E-cadherin. Since full-length CDCP1 does not bind E-cadherin, we did not expect enforced E-cadherin expression to alter TA inhibition of CDCP1 cleavage positively or negatively. To test this, we examined if enforced expression of E-cadherin or an E-Cadherin-Green Fluorescent Protein (GFP) fusion protein influences TA blockade of CDCP1 cleavage (Figure 2B). TA (0.5% w/v) decreased CDCP1 cleavage in MDA-MB-231 cells irrespective of E-cadherin overexpression and did not significantly impact CDCP1 tyrosine phosphorylation on multiple sites previously implicated in regulating downstream signaling proteins including Src (He et al., 2010; Liu et al., 2011). Consistent with this, TA did not alter Src phosphorylation on activating (Y416) or inhibitory (Y527) sites (Figure 2B). FAK and CDCP1 exhibit reciprocal tyrosine phosphorylation patterns in which FAK is heavily phosphorylated in cells attached to substrata, while CDCP1 is heavily phosphorylated in cells growing in suspension (Wortmann et al., 2011). TA did not alter FAK phosphorylation on the Y397 site that reflects FAK activation (Thamilselvan et al., 2007). Both the full length, 135 kDa, and cleaved, 85 kDa, forms of CDCP1 exhibited tyrosine phosphorylation at multiple sites.

We also screened the effects of TA on several other signaling pathways important in tumor growth and progression including the oncogenic transcription factors STAT3 and MYC, the mitogen-activated serine/threonine kinases S6 protein kinase (S6K), ERK, and AKT, and the levels and phosphorylation status of proteins that control cell cycle progression including Cyclin D1, Cyclin B1, and cleavage of PARP and Caspase 3 as markers of apoptotic cell death. TA reduced STAT3 tyrosine phosphorylation on its activating site, Y705, decreased MYC protein expression, and suppressed S6K1 phosphorylation on the activating site, T389 (Figure 2C). TA reduction of cell viability in MTT assays closely paralleled suppression of proliferation in thymidine incorporation assays (see Figures 1B,C). Consistent with this observation, TA did not increase the apoptotic markers Cleaved Caspase 3 (CC3) or PARP cleavage. However, tcyDTDO increased CC3 to detectable levels and elevated PARP cleavage as expected. TA had no consistent effect on ERK or AKT phosphorylation, Cyclin D1 or Cyclin B1 levels, or PARP or Caspase 3 cleavage across the MDA-MB-468, BT474, and WM793 human cancer cell lines (Supplementary Figure S1B).

TA treatments were also carried out in the presence of 10X excesses of Lys or Arg to examine the ability of high levels of these amino acids to overcome TA effects. High Lys or Arg levels did not alter STAT3 phosphorylation, but increased S6K1 phosphorylation and MYC levels consistent with the sensitivity of S6K1 activation (Iiboshi et al., 1999; Hidayat et al., 2003) and MYC expression (Yue et al., 2017) to amino acid levels (Figure 2C). However, TA still reduced S6K1 phosphorylation and MYC levels in the presence of excess Lys or Arg. Treatment with the protein synthesis inhibitor cycloheximide (CHX) was included as a control to determine if inhibition of protein synthesis was sufficient to mimic any TA effects. CHX decreased STAT3 phosphorylation, but did not decrease MYC levels or S6K1 phosphorylation. Control protein synthesis experiments showed that 1.25 μM CHX inhibits protein synthesis by approximately 60% (Supplementary Figure S1C).

Our group identified a class of compounds with anticancer activity termed DDAs that interfere with disulfide bond formation, induce endoplasmic reticulum (ER) stress, and inhibit protein synthesis (Ferreira et al., 2015; Ferreira et al., 2017; Wang et al., 2019a; Wang et al., 2019b). Results posted in a preprint suggest that DDAs act by inhibiting a subset of Protein Disulfide Isomerases (Law et al., 2021). Therefore, we examined if DDAs induce overlapping responses with TA and therefore may be useful in combination regimens against cancer. The DDA tcyDTDO decreased STAT3 and S6K1 phosphorylation and MYC levels (Figure 2C). Combined tcyDTDO/TA treatment reduced overall S6K1 phosphorylation more than either treatment alone as evidenced by electrophoretic mobility shifts of both the 70 and 85 kDa forms of S6K1 (Figure 2C, expanded region). We next examined the effect of TA on the phosphorylation of STAT3 and S6K1 in the MCF10A human mammary epithelial cell line overexpressing MYC and the MDA-MB-468 breast cancer line. TA induced a concentration-dependent decrease in STAT3 phosphorylation on Y705 but had no effect on phosphorylation on S727 (Figure 2D). The AGC-family kinases AKT and S6K1 have similar Arg-rich consensus substrate recognition sequences. Immunoblot analyses using antibodies recognizing phospho-AKT substrates and the S6K1 substrate S6 showed a TA-induced decrease in phosphorylation at these sites. This was paralleled by reduced phosphorylation of the S6K1 site S411 that is also associated with kinase S6K1 activation (Han et al., 1995). Together, the results discussed thus far in Figure 2 indicate that TA inhibits a subset of mitogenic/oncogenic signaling pathways. Quantification studies demonstrated that 2% w/v TA decreases phosphorylation of S6K1 on T389 and STAT3 on Y705, which are sites required for their kinase and transcriptional activities, respectively (Figure 2E). TA reduced CDCP1 cleavage in a concentration-dependent and statistically significant manner (Figure 2E). TA also decreased MYC expression levels, but this trend did not reach statistical significance.

### TA Inhibits Protein Synthesis and Reduces Histone Acetylation

**FIGURE 3:**
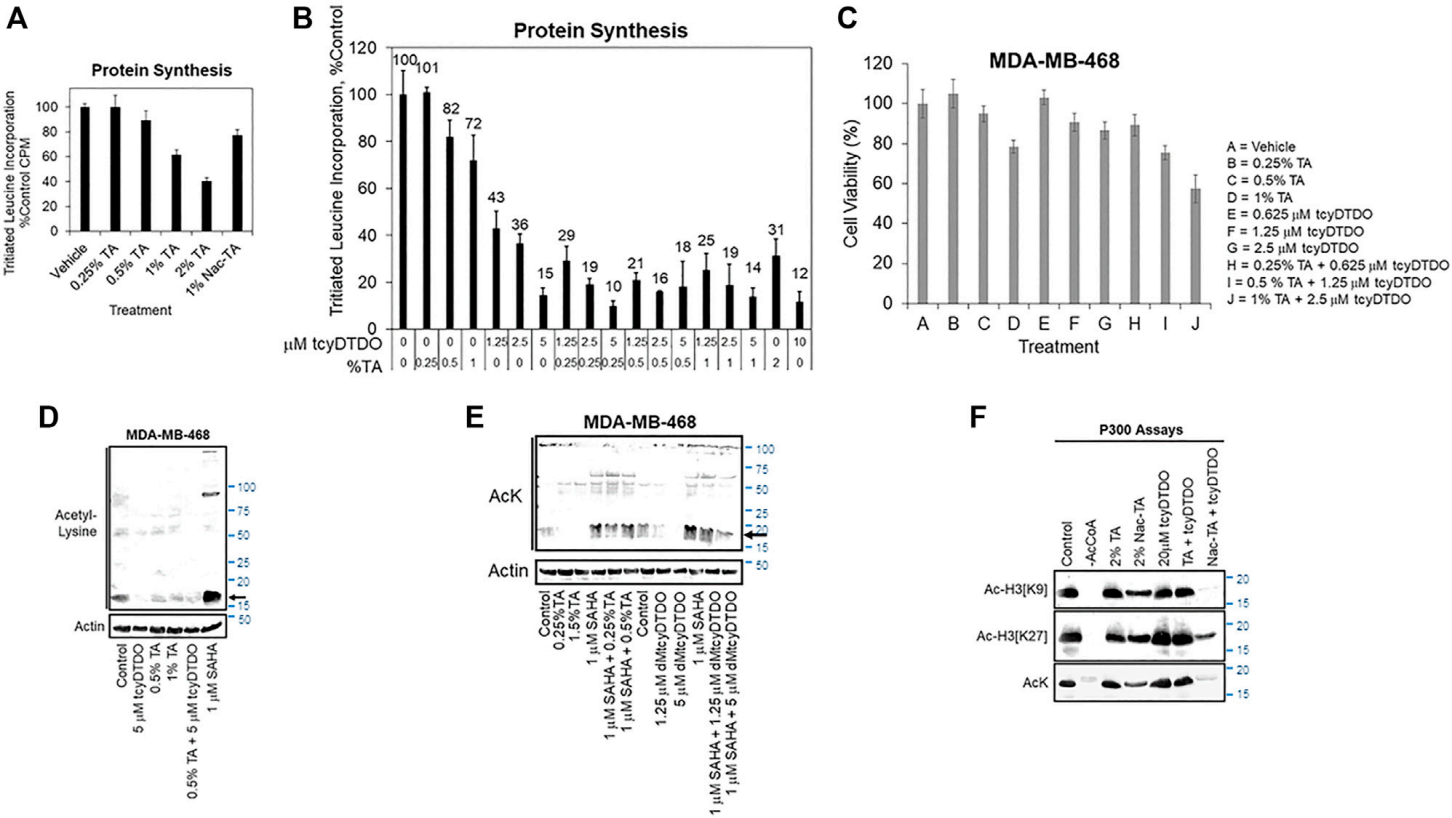
TA and DDAs exhibit partially overlapping anticancer mechanisms that include inhibition of protein synthesis, cell viability, and Histone acetylation. **(A)** Protein synthesis assay, performed as in Supplementary Figure S1C, examining the effects of 24 h treatments with varying TA concentrations, or the *N*-acetylated TA metabolite, NacTA, on protein synthesis in MDA-MB-468 cells. Results are the average of six replicates and presented as the average ±S.D. These findings are representative of three biological replicates with similar results. **(B)** MDA-MB-468 cells were treated for 24 h with the indicated concentrations of tcyDTDO and TA and protein synthesis assays were performed. Results are the average of six replicates and presented as the average ±S.D. These findings are representative of three biological replicates with similar results. **(C)** MTT cell viability assays performed after 72 h of the indicated treatments. Results are the average of six replicates and presented as the average ±S.D. These findings are representative of three biological replicates with similar results. **(D)** MDA-MB-468 cells were treated for 24 h as indicated and cell extracts were analyzed by immunoblot with antibodies recognizing acetyl-Lys (AcK) and Actin as a loading control. SAHA, also referred to as Vorinostat, is a Histone Deacetylase (HDAC) inhibitor. **(E)** MDA-MB-468 cells were treated for 24 h as indicated and cell extracts were analyzed by immunoblot with antibodies recognizing acetyl-Lys (AcK) and Actin as a loading control. The black arrow indicates the band corresponding to acetylated histones. **(F)** *In vitro* acetyltransferase assays employing p300 as the acetyltransferase and Histone 3 as the substrate. Assays were performed for 60 min at 37°C with the indicated additions and reaction mixtures were analyzed by immunoblot with the indicated antibodies recognizing acetyl-Lys or Histone 3 specifically acetylated on Lys residues 9 or 27. No acetylation was observed if Acetyl CoA was omitted from the reactions.

Since TA and protein synthesis inhibition by CHX both decreased STAT3 tyrosine phosphorylation (Figure 2C) and TA mimics Lys and Arg, we hypothesized that TA may inhibit protein synthesis. TA inhibited protein synthesis in a concentration-dependent manner, with 2% TA reducing protein synthesis by 60% (Figure 3A). DDAs also inhibit protein synthesis (Ferreira et al., 2017; Wang et al., 2019a; Wang et al., 2019b). Therefore, we examined if combining low concentrations of the DDA tcyDTDO and TA would suppress protein synthesis in an additive manner. Treatment with 1.25 μM tcyDTDO inhibited protein synthesis by 57% and addition of increasing concentrations of TA further reduced protein synthesis (Figure 3B). Similarly, combining concentrations of TA and tcyDTDO that have little effect on cancer cell viability alone resulted in larger reductions in viability (Figure 3C).

Suppression of histone acetylation by the p300/CBP enzymes is an emerging strategy for cancer therapy (Yang et al., 2013; Gu et al., 2016; Lasko et al., 2017; Wang et al., 2017; van Gils et al., 2021). We hypothesized that TA may alter Lys acetylation through competitive inhibition of Lys acetyltransferases and next examined if TA reduces acetylation of proteins on Lys. TA at 0.5 or 1% partially blocked protein Lys acetylation of a prominent ∼20 kDa band as detected with an acetyl-Lys antibody (Figure 3D, arrow). This band is likely acetylated histones, which are the most abundant acetylated cellular proteins in this molecular size range (Hansen et al., 2019). TcyDTDO also reduced acetylation of this band, and acetylation was strongly increased by treatment of the cells with the histone deacetylase inhibitor Vorinostat/SAHA. We next examined if TA could overcome the effects of SAHA. TA treatment reduced Lys acetylation, but TA did not override the increased acetylation induced by SAHA co-treatment (Figure 3E). In contrast, treatment with the DDA dMtcyDTDO (Law et al., 2021) decreased baseline Lys acetylation and blocked the SAHA-driven increase in acetylation. The p300 protein is a major cellular histone acetyltransferase (HAT). We next performed HAT enzyme assays to determine if TA or tcyDTDO directly inhibit p300 activity. TA (2%) did not affect p300 acetylation of Histone 3 *in vitro* (Figure 3F). TcyDTDO also did not directly inhibit p300 activity. TA is partly metabolized in the body by acetylation (0.5% of oral dose; http://www.accessdata.fda.gov/drugsatfda_docs/label/2020/022430s009lbl.pdf), and perhaps also in cultured cells. Therefore, we examined if the metabolite *N-*acetyl-TA (NacTA) alters p300 activity. NacTA partially inhibited p300 HAT activity and interestingly, combining NacTA and tcyDTDO strongly inhibited overall p300 activity (AcK), as well as acetylation of Histone H3 on Lys residues 9 and 27. Further studies are required to determine if TA, NacTA, or DDAs reduce cellular protein acetylation through effects on p300 or other HAT enzymes. However, the results in Figures 3D–F suggest that TA and/or its metabolites and DDAs may mediate their effects in part by altering protein acetylation.

### Efficacy of TA/DDA Mono- and Combination Anti-Cancer Therapy

**FIGURE 4:**
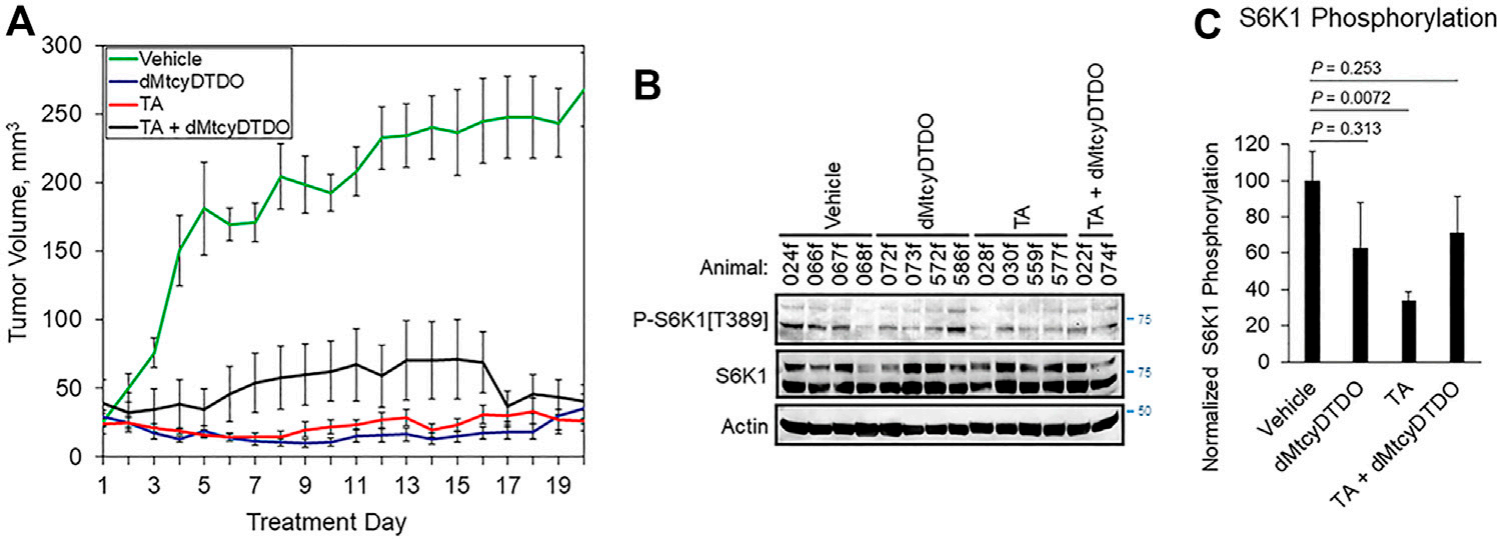
TA blockade of breast tumor growth is associated with reduced S6K1 phosphorylation. **(A)** Growth curves of 012/LVM2/LR10 tumors treated once daily by oral gavage with vehicle (peanut oil), 10 mg/kg dMtcyDTDO, 375 mg/kg TA or combined treatment with the 10 mg/kg dMtcyDTDO and 375 mg/kg TA. Results are the average obtained from 10 animals in each treatment group and presented as the average ±S.D. **(B)** Immunoblot analysis of tumor extracts with the indicated antibodies. **(C)** Plot of S6K1 phosphorylation normalized to S6K1 protein levels. Results represent the average of four samples from each treatment group. Error bars represent S.E.M. Pairwise statistical comparisons between the vehicle and drug treatment groups were made using unpaired Student’s *t*-tests.

The findings presented in Figures 1–3 indicated that TA suppressed the growth of tumors derived from 012/LVM2/LR10 cells in a 12-day study, and cell culture studies showed that TA may suppress cancer cell viability through multiple mechanisms. The results further suggest that TA biochemical mechanisms of anticancer action partially overlap with those of DDAs. Therefore, we examined the anticancer efficacy of 375 mg/kg TA, 10 mg/kg of the DDA dMtcyDTDO, or TA + dMtcyDTDO administered once daily by oral gavage in longer-term studies. Tumors were initiated by orthotopic injections of 10^6^ 012/LVM2/LR10 cells and treatment was initiated when tumors were detectable by palpation. By 20 days of treatment, tumors in vehicle-treated mice averaged over 200 mm^3^, while tumors in drug-treated animals were less than 50 mm^3^ (Figure 4A). Immunoblot analysis of tumor extracts from each group showed that TA reduced S6K1 phosphorylation on Thr^389^, which is required for its kinase activity (Dennis et al., 1998; McMahon et al., 2002), to a greater extent on average than the other treatments (Figure 4B). Quantification of the results in Figure 4B indicated that TA suppressed S6K1 phosphorylation in a statistically significant manner (Figure 4C). Rapamycin analogs (“rapalogs”) are in clinical use against human cancer and act in part by inhibiting S6K (Ferrari et al., 1993; Meng and Zheng 2015), and S6K inhibitors are under development as cancer therapeutics (Pearce et al., 2010; Nam et al., 2019), consistent with the well-established role of S6K activation in the molecular pathogenesis of multiple cancers.

**FIGURE 5:**
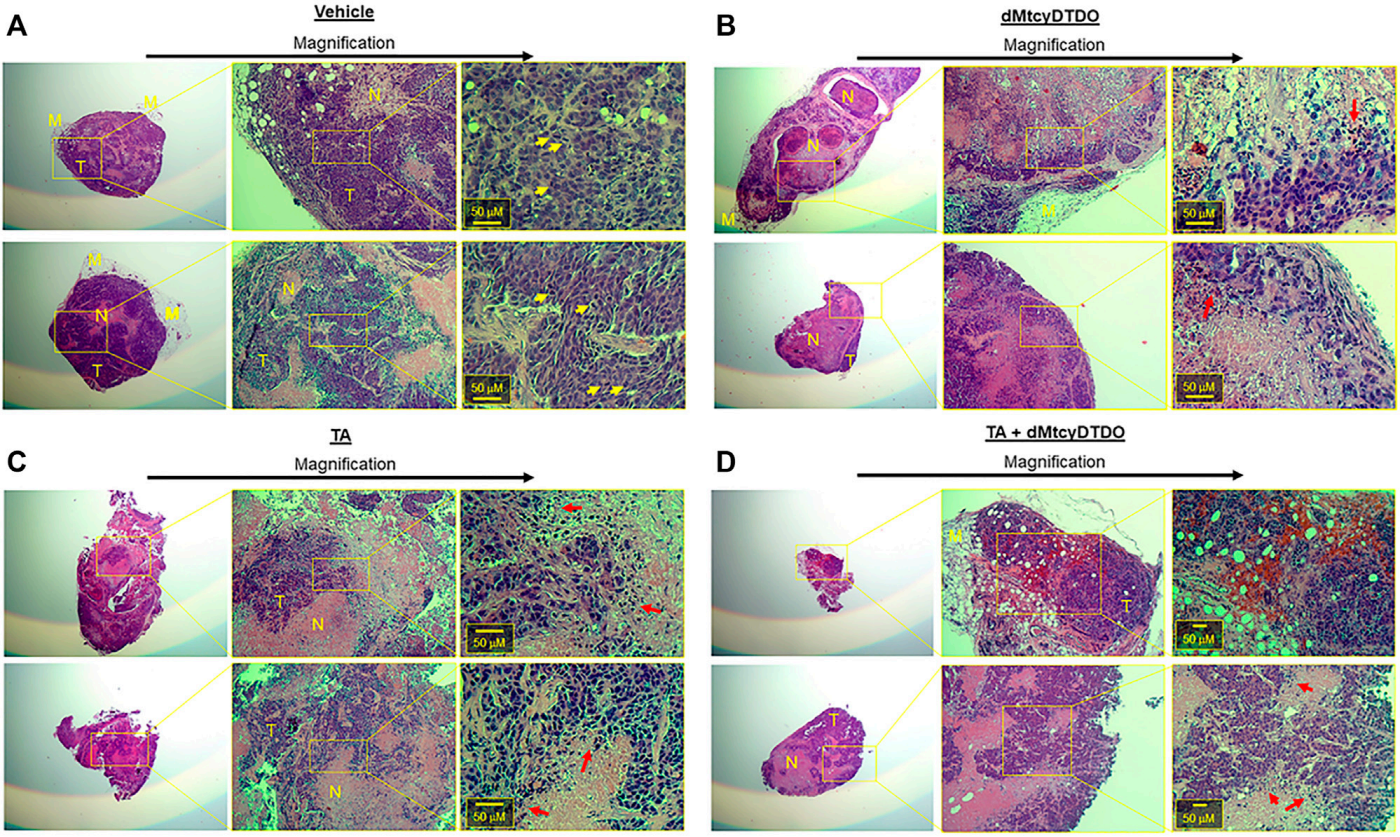
TA and dMtcyDTDO cause tumor cell death in animal models. At endpoint, two tumors from the **(A)** Vehicle, **(B)** dMtcyDTDO, **(C)** TA, and **(D)** TA + dMtcyDTDO treatment groups were paraffin-embedded, sectioned and stained with hematoxylin and eosin (H&E). Each section is shown at three different magnifications with scale bars on the highest magnification images. T refers to viable tumor tissue, N, necrotic/dead tissue, and M, mammary tissue. Yellow arrows denote cells undergoing mitosis and red arrows indicate cells undergoing nuclear condensation and fragmentation, indicative of cell death.

We previously observed that treatment with the DDA tcyDTDO caused widespread apoptosis of cancer cells in primary tumors and metastases without inducing apoptosis in adjacent normal tissues, resulting in substantial regions of dead tumor tissue (Wang et al., 2019b). This indicates that calculating tumor volumes without taking into account viability of the cancer cells may underestimate the efficacy of anticancer regimens. Further, sampling portions of such heterogeneous tumors may introduce errors into downstream proteomic or transcriptomic analyses. Therefore, we examined the morphology of Hematoxylin and Eosin (H&E)-stained tumors from each of the four treatment groups. Two representative tumors from vehicle-treated mice (Figure 5A) exhibited predominantly viable tumor tissue (T), with small regions of dead cancer cells (N). Higher magnification images showed mitotic figures, demonstrating cancer cell proliferation in the vehicle-treated tumors (yellow arrows). Representative tumors from dMtcyDTDO-treated animals in contrast, exhibited predominantly dead tumor tissue (N), with small areas of apparently viable tumor cells (T) (Figure 5B). Closer inspection of the boundary between the dead and viable cancer cells showed tumor cells undergoing nuclear condensation and fragmentation (red arrows), consistent with apoptosis. Interestingly, tumors from TA-treated animals exhibited somewhat similar morphology to the tumors from dMtcyDTDO-treated animals with large regions of dead tumor tissue and tumor cells exhibiting nuclear condensation and fragmentation (red arrows, Figure 5C). Tumors from mice treated with TA + dMtcyDTDO (Figure 5D) exhibited similar morphology to the tumors from mice treated with TA or dMtcyDTDO with large regions of dead tumor tissue, tumor cells exhibiting nuclear condensation and fragmentation, and a lack of mitotic figures. A few previous studies have noted TA anticancer activity in xenograft models (Astedt and Trope 1980; Iwakawa and Tanaka 1982; Ogawa et al., 1982), and in a case report (Astedt et al., 1977), against several solid tumor types, including breast cancer. In these reports, TA anticancer activity was attributed to its antifibrinolytic actions. Our results demonstrate TA efficacy against breast tumors in animal models and identify several novel TA-induced biochemical responses that may contribute to TA anticancer activity.

### TA Suppression of Arg and Lys Absorption by Cancer Cells

**FIGURE 6:**
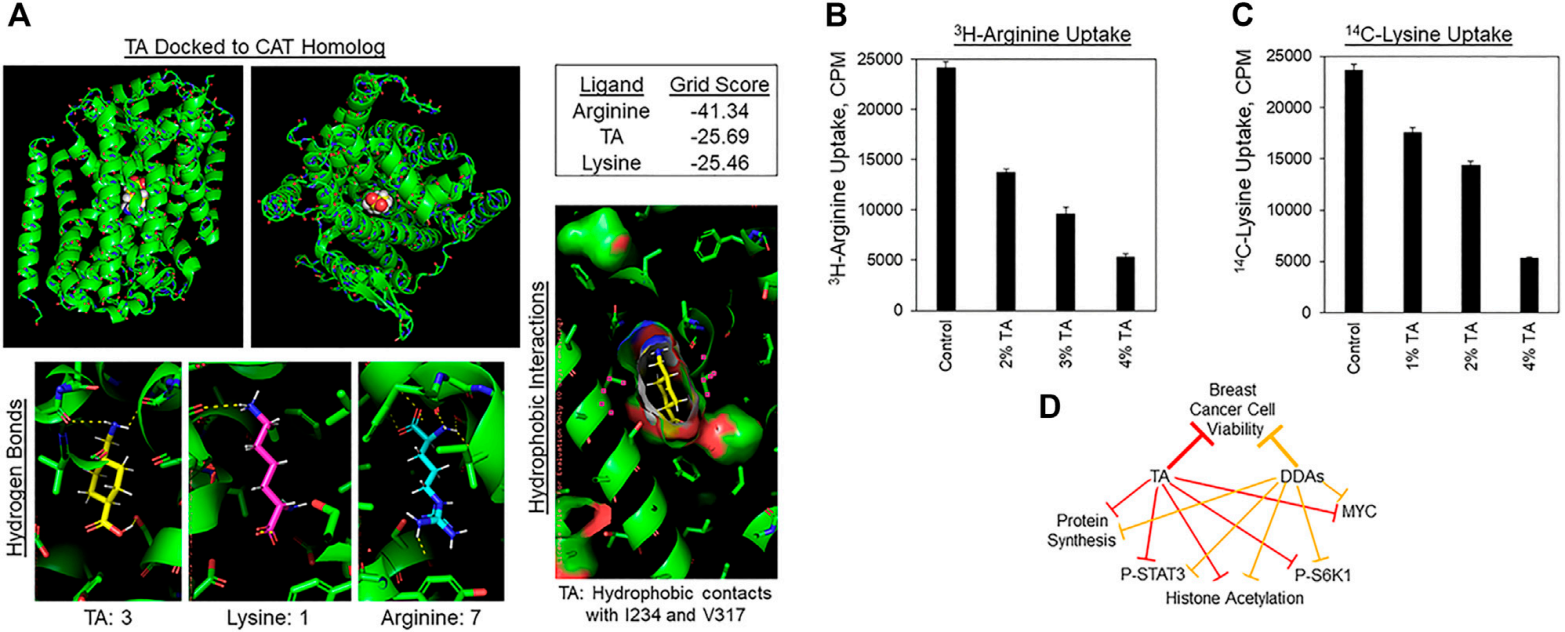
TA inhibits Lys and Arg absorption by cancer cells. **(A)** Molecular docking of TA into the Arg binding pocket of the bacterial homolog of the Cationic Amino acid Transporters (CATh). Upper-left: Image of TA docked to CATh in the plane of the plasma membrane. Upper-middle: View of TA docked into CATh orthogonal to the membrane. Upper-right: Binding energy scores for Arg, TA, or Lys binding to the Arg binding pocket of CATh estimated by DOCK 6.7. Lower-left: Hydrogen bonds between CATh and TA, Lys, or Arg predicted by PyMol. Lower-right: Hydrophobic contacts between I234 and V317 of CATh with the cyclohexane ring of TA predicted by PyMol. **(B)** ^3^H-Arg uptake by MDA-MB-468 cells during a 2 h labeling pulse in the presence of the indicated concentrations of TA. Results are the average of six replicates and presented as the average ±S.D. These findings are representative of three biological replicates with similar results. **(C)** ^14^C-Lys uptake by MDA-MB-468 cells during a 2 h labeling pulse in the presence of the indicated concentrations of TA. Results are the average of six replicates and presented as the average ±S.D. These findings are representative of three biological replicates with similar results. **(D)** Model displaying the overlapping biochemical mechanisms by which TA and DDAs may suppress breast tumor growth.

As mentioned above, S6K1 activation is sensitive to the levels of amino acids, as is MYC expression and the initiation of protein synthesis. Given the similarity of TA to Lys and Arg, we hypothesized that TA may compete with Lys or Arg for uptake by Cationic Amino acid Transporter (CATs). If correct, this hypothesis may also explain how TA enters cells since TA contains charged carboxyl and amino groups and is thus unlikely to traverse the plasma membrane unaided. The structure of a bacterial homolog of the mammalian CAT-family proteins (CATh) has been obtained with Arg bound (Jungnickel et al., 2018). We deleted Arg from this structure to perform *in silico* molecular docking to determine if TA is predicted to bind to this pocket, and if so, how its predicted binding energy compares with that of Arg or Lys. Figure 6A, upper-left and upper-middle shows a model of how TA might bind to CATh as viewed in the plane of the plasma membrane, or looking down into the transporter pore. The DOCK 6.7 program estimated Arg to have the highest binding energy for the CATh pocket, followed by TA, and Lys. It must be noted that if CATh bound to Lys or TA had served as the original model rather than CATh bound to Arg the results may have differed. However, the results obtained suggest that TA might bind to CATh with an affinity that is similar in magnitude with that of Arg and Lys. We next examined if TA suppresses the uptake of radiolabeled Arg or Lys by breast cancer cells. TA reduced the absorption of ^3^H-Arg by MDA-MB-468 cells in a concentration-dependent manner (Figure 6B). Similarly, TA decreased ^14^C-Lys uptake by MDA-MB-468 cells in a concentration-dependent manner (Figure 6C). These results suggest that TA may reduce cancer cell viability in part by inhibiting import of Lys and Arg through competition for CATs or other amino acid transporters, and that TA might enter cancer cells through these same transporters.

## Discussion

Previous work suggested that TA might exhibit anticancer activity by inhibiting Plasmin activity and by preventing Plasminogen conversion to Plasmin (O'Grady et al., 1981; Tanaka et al., 1982; Stonelake et al., 1997; Dunbar et al., 2000; Suojanen et al., 2009). The results presented here indicate that TA blocks cleavage of the Plasmin substrate CDCP1. Some reports indicate that the cleaved form of CDCP1 exhibits enhanced oncogenic functions and a different spectrum of protein-protein interactions than the full-length protein (Casar et al., 2014; Law et al., 2016; Wright et al., 2016). Suppression of CDCP1 cleavage by TA may be preferable to that induced by TGFβ and glucocorticoids since these agents block Plasminogen activation by upregulating Plasminogen Activator Inhibitor-1 (PAI-1) and activate transcriptional programs that may not be beneficial in the cancer setting. However, blockade of Plasminogen activation and cleavage of CDCP1 and other Plasmin substrates represents only one of the biochemical actions of TA that could contribute to its anticancer activity.

The MYC and STAT3 oncoproteins are widely overexpressed or activated across human cancers and the mTORC1/S6K1 pathway is frequently stimulated in malignancies. Of interest is the observation that MYC transformed MCF10A mammary epithelial cells are more sensitive to TA-mediated reduction in cell viability than vector control cells. This may be due to MYC-mediated monopolization of the protein translation machinery and regulation of the translation factors eIF4E, eIF4A, and eIF4G (Ruggero 2009; Flynn and Hogarty 2018) and could explain in part how TA suppresses breast tumor growth in animal models without evidence of toxicity. Given these observations, more work is warranted to elucidate the biochemical mechanisms by which TA suppresses MYC expression, and STAT3 and S6K1 phosphorylation, and reduces histone acetylation. The protein synthesis inhibitor Cycloheximide partially blocked STAT3 tyrosine phosphorylation (Figure 2C), but did not reduce MYC levels or S6K1 phosphorylation, indicating that the latter two TA effects are independent of protein synthesis inhibition.

TA inhibition of protein synthesis is of particular interest because Asparaginase, which depletes asparagine and glutamine, is in clinical use against leukemia (reviewed in Nikolaev 1969) and functions in part by suppressing protein synthesis (Ellem et al., 1970; Kessel and Bosmann 1970; Benvenisty 1972) and by inhibiting mTORC1/S6K1 signaling (Nikonorova et al., 2018). Asparaginase resistance remains a significant clinical problem (Apfel et al., 2021) and as a protein drug, Asparaginase suffers from issues associated with instability and rapid clearance (Varshosaz and Anvari 2018) and allergenicity (Belen et al., 2019). Thus, a small molecule drug such as TA or a TA analog may be useful against Asparaginase-resistant malignancies or in patients with Asparaginase allergies. Examining TA effects on Lys/Arg absorption by cancer cells showed inhibition of uptake of both amino acids. Depletion of cellular Lys/Arg could explain TA-induced suppression of protein synthesis and reduced S6K1 phosphorylation (Drummond et al., 2008). Since solid tumors are expected to be nutrient-deprived in their avascular centers, the consequences of TA suppression of Lys/Arg uptake may be amplified in the tumor microenvironment. Amino acid levels also regulate the process of autophagy, which may mediate cell death depending on the circumstances. Future studies should examine if TA treatment stimulates autophagy, and if autophagy contributes to the anticancer actions of TA.

Results posted in a preprint suggest that DDAs may suppress tumor growth by inhibiting a subset of Protein Disulfide Isomerases (PDIs) (Law et al., 2021). Interestingly, DDAs exhibit some activities that overlap with those of TA, including decreased MYC expression, sensitivity enhancement by MYC overexpression, lowering of STAT3 tyrosine phosphorylation and S6K1 phosphorylation on Thr^389^ and Ser^411^, inhibition of protein synthesis, and reduced histone acetylation. Figure 6D summarizes the common biochemical responses to TA and DDAs that are hypothesized to mediate in part, the anticancer activity of these compounds. The results in Figure 4 examining TA/DDA mono- and combination regimens against breast tumors indicate that TA suppresses S6K1 phosphorylation on Thr^389^ in tumors *in vivo*.

TA exhibits anticancer activity in mice at a dosage of 375 mg/kg (Figures 1F, 4A). Future studies are required to determine whether similar TA anticancer activity is observed at lower dosages. Clinically, TA has been used at dosages of 20 mg/kg to suppress postoperative bleeding (Andersson et al., 1968). The most common intravenous dosages used clinically are 10–20 mg/kg, given every 6–8 h, or simply before and after surgery (Lecker et al., 2012). Administration of 240 mg/h TA was used to prevent life-threatening bleeding in advanced cancer patients (Atreya 2021). However, dosages as high as 100 mg/kg followed by 10 mg/kg/hour were given previously, until it was found that total doses of 80–100 mg/kg led to a small increase in post-operative seizures. Seizures are an important side effect of TA when used at high dosages (Mohseni et al., 2009; Kalavrouziotis et al., 2012). This is thought to result primarily from TA induction of neuronal hyperexcitability due to antagonism of glycine binding to glycine receptors, which hyperpolarize neurons (Lecker et al., 2012). Importantly, this neuronal hyperexcitability may be overcome using the common surgical anesthetics isoflurane and propofol (Lecker et al., 2012). In addition, seizures have been observed almost only following cardiac surgery, and this effect may not be applicable or as significant in systemic treatment of non-surgical cancer patients.

TA side effects may also be avoided by using it against skin cancers via topical administration or against other surface-accessible malignancies such as superficial bladder cancers (Javadpour 1989) or lung tumors (Moghissi et al., 2004; Jang et al., 2006). Although a 1% (w/v) TA concentration is 64 mM, this concentration is in line with established topical or intradermal concentrations administered for bleeding reduction (0.7%, (Ker et al., 2013)), melasma (3%, (Ebrahimi and Naeini 2014)), or rosacea (5–10%, (Jakhar et al., 2020; Daadaa et al., 2021)). In addition, it has been well established that topically administered TA has less than 10% of the systemic impact of a similar dose delivered intravenously (Ker et al., 2013; Ausen et al., 2019). Given the small volumes that are likely to be administered topically, the risk of seizure would not appear to be an issue.

It should be noted that while TA is not currently approved for use as an anticancer agent, TA is employed with increasing frequency in various aspects of clinical cancer management. TA is often used to limit bleeding during surgical tumor resections (Wu et al., 2006; Longo et al., 2018; Jaffer et al., 2021). TA treatment is associated with a reduction in the number of malignant cells and decreased generation of ascites fluid in patients with disseminated ovarian cancer (Soma et al., 1980; Kikuchi et al., 1986). In the treatment of node-positive, operable breast cancer, the axillary lymph nodes are dissected out, and this is associated with excessive axillary drainage and risk of infection. TA treatment reduces axillary drainage and decreases the rate of complications in these patients (Oertli et al., 1994; Lohani et al., 2020; Lohani et al., 2021). It will be important to determine in these clinical scenarios where TA is used to control treatment-associated morbidities, if TA also contributes to improved patient outcome through direct actions against any remaining cancer cells. If so, then in cancers such as Triple-Negative Breast Cancer (TNBC), which exhibit high rates of post-surgical recurrence (Pilewskie et al., 2014; Radosa et al., 2017), TA treatment before or after tumor resection might reduce the rate of cancer recurrence.

The beneficial actions of TA in inhibiting Plasmin activation and activity in the cancer setting are well appreciated, but to our knowledge, the present study is the first to survey the effects of TA on an array of other signaling pathways and proteins with roles in tumor growth and progression. The different pharmacological mechanisms of action exhibited by TA may be explained by the ability of TA to antagonize Lys and Gly binding sites, and perhaps also Arg and His binding sites. Additional work is needed to elucidate how TA mediates its Plasmin-independent signaling effects. Additionally, TA is anti-inflammatory and modulates the innate immune system (Draxler et al., 2019). This study by Draxler et al. was performed alongside the major prospective safety study of TA, ATACAS, where they found that use of TA did not cause an increase in thromboembolic events in the only prospective study with sufficient power to prove this point. The Draxler adjunctive study found that the patients who received TA had significantly fewer post-operative infections, despite the fact that every patient received prophylactic antibiotics. Draxer et al. also administered a standard dose of TA to healthy volunteers. In both the healthy volunteers and the surgical patients they found that TA upregulated multiple immune-enhancing markers and downregulated multiple immunosuppressive markers.

We hypothesize that the multiple anticancer activities of TA may delay or block the acquisition of tumor resistance, and this might be further enhanced by combining TA with other classes of anticancer agents and/or therapies. It will also be important in future studies to determine if TA metabolites such as NacTA play significant roles in the responses of cancer cells to TA. The observation that TA elicits multiple biochemical responses that are expected to block tumor growth and progression suggests that it may be possible to design new TA analogs that more potently or selectively trigger individual downstream TA anticancer actions. Specifically, TA analogs may be designed that do not antagonize the glycine receptor and cause seizures, and are more potent inhibitors of Arg and Lys transporters.

## Data Availability

The original contributions presented in the study are included in the article/Supplementary Material, further inquiries can be directed to the corresponding author.
