# Supplementary material for: Repurposing Tranexamic Acid as an Anticancer Agent
Source: Front Pharmacol. 2022 Jan 13;12:792600. doi: 10.3389/fphar.2021.792600 (PMC8793890; doi:10.3389/fphar.2021.792600)
Supplement: Supplementary file 1 [file DataSheet1.PDF]

Supplementary Material

Repurposing Tranexamic Acid as an Anticancer Agent

Mary E. Law, Bradley J. Davis, Amanda F. Ghilardi, Elham Yaaghubi, Zaafir M. Dulloo, Mengxiong Wang, Olga Guryanova, Coy D. Heldermon, Stephan C. Jahn, Ronald K. Castellano, and Brian K. Law

Table of Contents | Pages

Supplemental Figure (S) 1:
TA inhibits DNA synthesis without affecting the levels of Cyclin B and Cyclin D1 or the phosphorylation of Akt, ERK and RB 2

Supplemental Table 1: Sources of antibodies 3

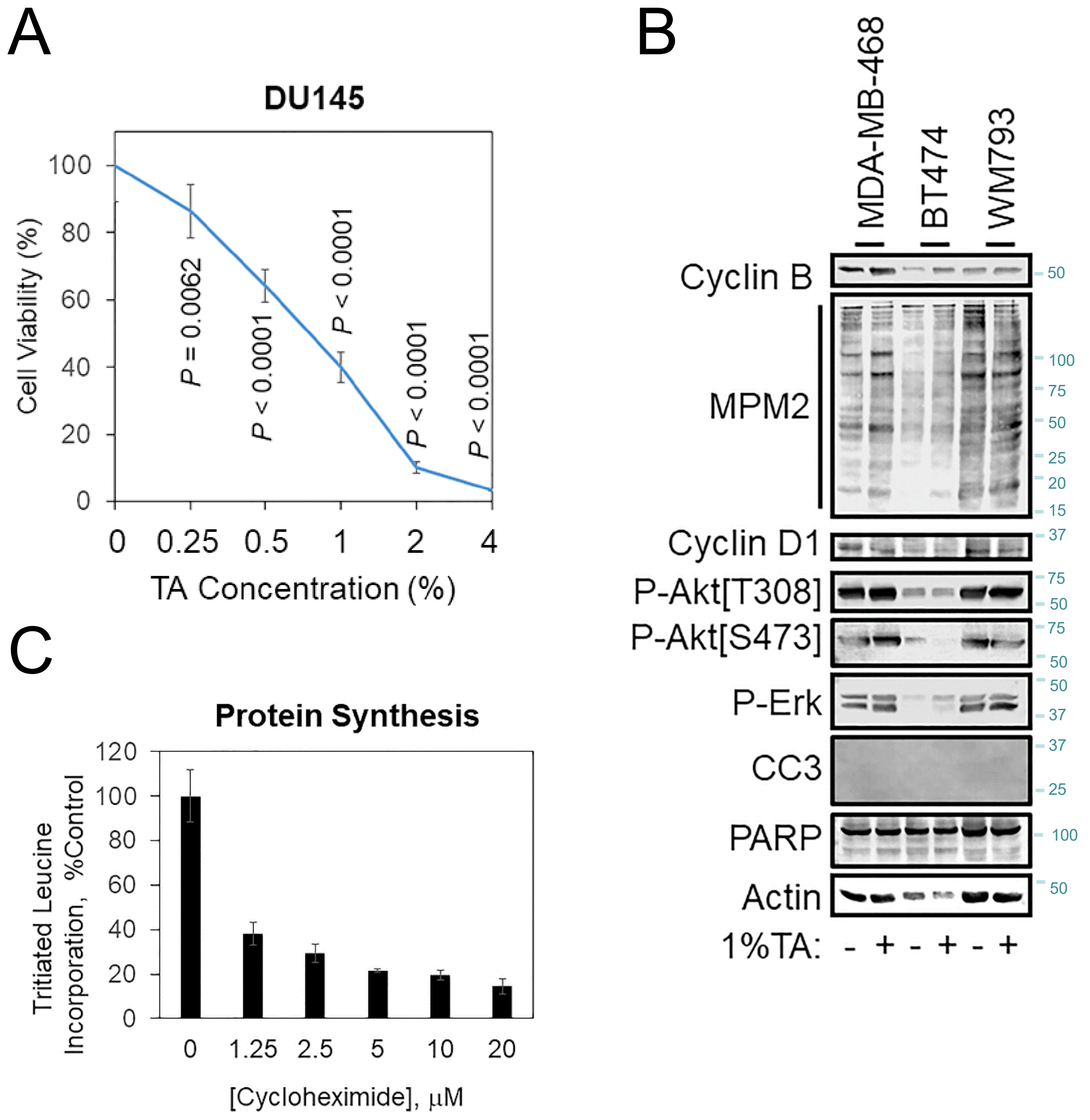

Figure S1: TA inhibits DNA synthesis without affecting the levels of Cyclin B and Cyclin D1 or the phosphorylation of Akt, ERK and RB. A. Viability assay on DU145 cells performed as in Fig. 1A. examining the statistical significance of TA induced reduction in cell viability. Statistically significant reductions in viability were observed at all TA concentrations tested, down to 0.25% w/v. Results are the average of six replicates and presented as the average ± S.D. These findings are representative of three biological replicates with similar results. B. The indicated cell lines were treated for 24 h with or without 1% TA and cell extracts were analyzed by immunoblot with the indicated antibodies. C. MDA-MB-468 cells were treated for 24 h as indicated and protein synthesis was assayed by tritiated Leu incorporation into protein. Note that 1.25 µM CHX reduces overall protein synthesis by approximately 60%. Results are the average of six replicates and presented as the average ± S.D. These findings are representative of three biological replicates with similar results.

Supplementary Material Table 1: Sources of antibodies used in immunoblot analyses.		
**Antibody**	**Catalog Number**	**Company**
Ac-H3[K9]	#9649	Cell Signaling Technology (Beverly, MA USA)
Ac-H3[K17]	#4353	Cell Signaling Technology (Beverly, MA USA)
AcK (Acetyl lysine	#9441	Cell Signaling Technology (Beverly, MA USA)
p-Akt Substrate (RXXS/T)	#9614	Cell Signaling Technology (Beverly, MA USA)
p-Akt[S473]	#9271	Cell Signaling Technology (Beverly, MA USA)
p-Akt[T308]	#13038	Cell Signaling Technology (Beverly, MA USA)
CDCP1	#13794	Cell Signaling Technology (Beverly, MA USA)
p-CDCP1[Y707]	#13111	Cell Signaling Technology (Beverly, MA USA)
p-CDCP1[Y734]	#9050	Cell Signaling Technology (Beverly, MA USA)
pCDCP1[Y743]	#13093	Cell Signaling Technology (Beverly, MA USA)
pCDCP1[Y806]	#13024	Cell Signaling Technology (Beverly, MA USA)
Cleaved Caspase 3	#9661	Cell Signaling Technology (Beverly, MA USA)
Cyclin B	#4135	Cell Signaling Technology (Beverly, MA USA)
p-ERK	#9101	Cell Signaling Technology (Beverly, MA USA)
p-FAK[Y397]	#8556	Cell Signaling Technology (Beverly, MA USA)
PARP	#9532	Cell Signaling Technology (Beverly, MA USA)
PD-L1	#13684	Cell Signaling Technology (Beverly, MA USA)
Rb	#9309	Cell Signaling Technology (Beverly, MA USA)
p-Rb[S780]	#9307	Cell Signaling Technology (Beverly, MA USA)
p-Rb[S795]	#9301	Cell Signaling Technology (Beverly, MA USA)
pRb[S807/811]	#9308	Cell Signaling Technology (Beverly, MA USA)
p-S6[S235/236]	#2211	Cell Signaling Technology (Beverly, MA USA)
p-p70 S6 Kinase[T389]	#9205	Cell Signaling Technology (Beverly, MA USA)
p-Src[Y416]	#6943	Cell Signaling Technology (Beverly, MA USA)
p-Src[Y527]	#2105	Cell Signaling Technology (Beverly, MA USA)
pSTAT3[Y705]	#9131	Cell Signaling Technology (Beverly, MA USA)
pSTAT3[Y727]	#9134	Cell Signaling Technology (Beverly, MA USA)
β-Actin	sc-47778	Santa Cruz Biotechnology (Santa Cruz, CA USA)
Cyclin D1	sc-450	Santa Cruz Biotechnology (Santa Cruz, CA USA)
Myc	sc-40	Santa Cruz Biotechnology (Santa Cruz, CA USA)
Myc	sc-42	Santa Cruz Biotechnology (Santa Cruz, CA USA)
p70 S6 Kinase	sc-230	Santa Cruz Biotechnology (Santa Cruz, CA USA)
Src	sc-18	Santa Cruz Biotechnology (Santa Cruz, CA USA)
STAT3	sc-7179	Santa Cruz Biotechnology (Santa Cruz, CA USA)
MPM2	05-368	Upstate Biotechnology (Lake Placid, NY USA)
E-Cadherin	#610182	BD Transduction Laboratories (San Jose, CA USA)
PAI-1	#612024	BD Transduction Laboratories (San Jose, CA USA)
